# Prognostic value of the systemic immune-inflammation index in critically ill elderly patients with hip fracture: evidence from MIMIC (2008–2019)

**DOI:** 10.3389/fmed.2024.1408371

**Published:** 2024-05-30

**Authors:** Zhen-Jiang Liu, Gen-He Li, Jing-Xuan Wang, Zhi-Hong Mo, Kang-Yong Yang, Chu-Long Shen, Zhao-Xiong Shen

**Affiliations:** ^1^The Eighth Clinical Medical College of Guangzhou University of Chinese Medicine, Foshan, Guangdong, China; ^2^Foshan Hospital of Traditional Chinese Medicine, Foshan, Guangdong, China

**Keywords:** systemic immune-inflammation index, critically ill elderly patients, hip fracture, prognostic value, mortality, MIMIC-IV database

## Abstract

**Background:**

The systemic immune-inflammation index (SII) showed an extensive link between immunological dysfunction and the activation of systemic inflammation. Several studies have confirmed the application of SII to orthopedic diseases. However, the significance of SII in critically ill elderly individuals with hip fracture who require intensive care unit (ICU) admission is not yet known. This study centered on exploring the relationship between SII and clinical outcomes among critically ill elderly hip fracture individuals.

**Methods:**

The study centered around elderly patients experiencing severe illness following hip fractures and requiring admission to the ICU. These patients from the MIMIC-IV database formed the basis of this study’s cohort. We stratified them into quartiles according to their SII levels. The results involved the mortality at 30 days and 1 year post-admission. Then we employ Cox proportional hazards regression analysis as well as restricted cubic splines to explore the association between the SII and clinical results in critically ill elderly patients with hip fracture.

**Results:**

The study encompassed 991 participants, among whom 63.98% identified as females. Notably, the mortality rates attributed to any cause within 30 days and 1 year after hospitalization stood at 19.68 and 33.40%, respectively. The multivariate Cox proportional hazards model disclosed a significant correlation between an elevated SII and all-cause mortality. Following adjustments for confounding variables, individuals with a high SII showed a notable correlation with 30-day mortality [adjusted hazard ratio (HR), 1.065; 95% confidence interval (CI), 1.044–1.087; *p* < 0.001] and 1-year mortality (adjusted HR, 1.051; 95% CI, 1.029–1.074; *p* < 0.001). Furthermore, the analysis of restricted cubic splines demonstrated a progressive increase in the risk of all-cause death as the SII value rose.

**Conclusion:**

Among critically ill elderly patients with hip fracture, the SII exhibits a non-linear association that positively correlates with both 30-day and 1-year all-cause mortality rates. The revelation indicates that the SII may play a vital role in identifying patients with hip fractures who face an escalated risk of mortality due to any cause.

## 1 Introduction

Hip fractures are common in individuals aged 70 years or older, and the incidence of hip fractures has risen by 93.0% from 1990 to 2019, with an estimated 14.2 million cases globally, which poses a significant threat to public health (1). Despite a considerable number of elderly adults receiving hospitalization post-injury, the risk of complications and mortality remains high. Approximately 9% of general patients succumb within a month, and this figure escalates to a staggering 36% for mortality within a year’s timeframe (2). Some researchers suggest that the elevated mortality rate of hip fractures is linked to the patient’s pathological and behavioral status, including nutritional deficiencies, immune system functioning, and the diminishment of skeletal muscle mass (3). Several studies have provided similar elucidation of the relationship between hip fracture mortality and immune health, particularly the inflammatory response (4). Published studies have demonstrated that the systemic immune-inflammation index (SII) functions as a distinct predictor of mortality subsequent to hip fracture incidents (5, 6). The SII is composed of the counts of neutrophils, lymphocytes, and platelets in peripheral blood, serving as a reliable indicator of inflammation. Furthermore, numerous studies have linked SII to predicting outcomes in various diseases among the general population and specific high-risk patient groups (6), for instance, malignancy (7), coronary artery disease (8), and acute ischemic stroke (9). Meanwhile, the SII has been progressively utilized for prognostication in orthopedics for preoperative evaluation (10), surgical trauma (11), and postoperative complications (12).

The SII provides a convenient and effective method for examining immunological dysfunction and the activation of systemic inflammation (13, 14). Prior research has mostly concentrated on evaluating SII levels in the general population to forecast adverse outcomes in hip fracture disease (3, 5). Several studies have found a significant link between high SII levels, inflammation advancement, and higher mortality rates for fractures (15). Moreover, multiple studies have demonstrated that SII is effective in forecasting the probability of post-surgical complications, including deep vein thrombosis (DVT), pneumonia, and even mortality in hip fracture patients (10, 16). Increasing evidence consistently connects high SII levels to elevated all-cause death rates. However, it is unclear whether this connection continues to exist in critically ill elderly individuals with hip problems who typically exhibit poorer pathophysiology. Therefore, evaluating the potential of SII in forecasting health complications among elderly individuals with severe hip conditions could aid in pinpointing those with an elevated risk of mortality from any cause, thereby facilitating timely medical intervention or emergency treatment.

The investigation sought to assess the prognostic value of the SII on overall mortality in aged critical patients with hip fracture, utilizing data sourced from the Medical Information Mart for Intensive Care IV (MIMIC-IV) database, considering the present state of scholarly research.

## 2 Methods

### 2.1 Data source

The research conducted a retrospective analysis of health-associated records sourced from the MIMIC-IV database, version 2.2, developed and managed by the MIT Computational Physiology Laboratory. The MIMIC-IV database encompasses numerical information related to the medical records of patients who were either admitted to the Intensive Care Unit (ICU) or received treatment in the emergency department at the Beth Israel Deaconess Medical Center, spanning the years between 2008 and 2019 (17). Authorization for Zhen-Jiang Liu, the first author (certification identifier: 12911307), was granted to access the MIMIC-IV database upon successful completion of the online educational program provided by the National Institutes of Health. The BIDMC Institutional Review Board evaluated the collection of patient data and the creation of the research resource. Then they gave approval to the data-sharing initiative and exempted the need for informed permission. All processes were executed in compliance with the regulations governing patient privacy and confidentiality.

### 2.2 Patient selection

**Figure 1:**
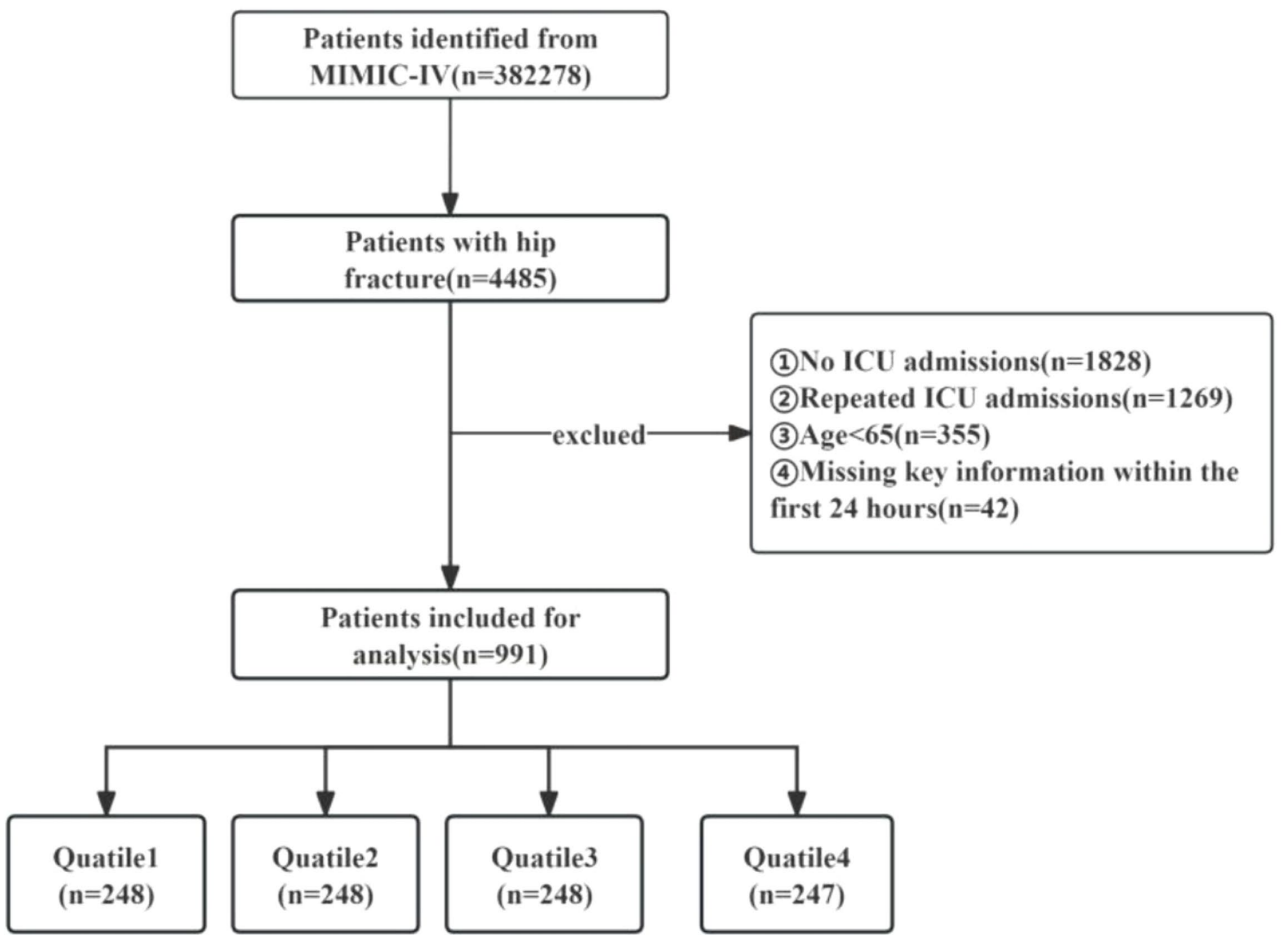
The flowchart of the patient selection process in the trial.

4,485 patients with hip fractures were identified in the MIMIC-IV database based on specific ICD codes: ICD-9:820, ICD-9:821, ICD-9:73314, ICD-9:73396, ICD-10:S72, ICD-10:S790, ICD-10:M9666, ICD-10:M8445, ICD-10:M8435, ICD-10:M8005, and ICD-10:M8085. The dataset encompasses a period ranging from January 2008 through December 2019. The eligibility criteria for inclusion were as follows: (I) participants aged 65 years or older; (II) those diagnosed with a hip fracture; and (III) patients necessitating ICU admission. Exclusion standards comprised the following: (I) instances where subjects had inadequate or untraceable documentation or pivotal medical records; (II) those who experienced multiple ICU admissions due to hip fracture, with only the initial admission data being considered; (III) individuals with missing survival outcome information; and (IV) patients devoid of critical data (neutrophil, lymphocyte, or platelet counts) on the day of admission. Ultimately, a collective of 991 participants were incorporated into the investigation and allocated into four categories in accordance with the quartiles of the SII (Figure 1).

### 2.3 Data collectioin

Data extraction was carried out utilizing PostgreSQL software (version 13.7.2) alongside Navicate Premium (version 16), which facilitated the process through the execution of Sequential Query Language (SQL) commands. The procurement of feasible variables encompasses five primary aspects: (1) basic demographics, including age, gender, height, body mass, and body mass index (BMI). (2) vital signs, such as body temperature, heart rate, respiratory rate, mean blood pressure, systolic blood pressure, diastolic blood pressure, and pulse oximetry-derived oxygen saturation (SpO2). (3) comorbidities, including diabetes mellitus, rheumatic disease, chronic obstructive pulmonary disease (COPD), deep venous thrombosis (DVT), pulmonary embolism (PE), dementia, coronary heart disease, osteoporosis, sepsis, chronic kidney disease, pneumonia, cerebral infarction, and hypertension. (4) laboratory indicators, including anion gap、CK-MB, BUN, ALP, bicarbonate, total bilirubin, sodium, chloride, calcium, potassium, PT, PTT, PT-INR, creatinine, WBC counts, lymphocyte counts¸neutrophil counts, platelet counts, monocyte counts, hemoglobin, hematocrit. (5) treatment: heparin, mechanical ventilation. (6) Admission severity of disease scores, including the Sepsis-related Organ Failure Assessment Score (SOFA), the simplified Acute Physiology Score II (SAPS-II), the Logistic Organ Dysfunction System (LODS), and the Oxford Acute Severity of Illness Score (OASIS) (18–21). The follow-up period commenced on the admission date and ended on the day of death. The computation of the SII adheres to this equation: SII = platelets count×neutrophils count÷lymphocytes count (22). All variables were collected from records obtained within the first day of the individual’s admission to the ICU.

Variables with more than 20% of their values missing were removed from the analysis to avert any prejudice. For variables with missing data below 20%, an imputation process was executed utilizing a random forest method, which was educated by the available non-missing variables. This procedure was carried out via the “mice” package within the R programming environment (Supplementary Table S1) (23).

### 2.4 Clinical outcomes

The primary outcomes for this investigation centered on the 30-day all-cause mortality rate following hospital admission, with the secondary outcome being the all-cause mortality rate within a year of admission.

### 2.5 Statistical analysis

Continuous data were depicted as mean ± SD if normally distributed. If not, they were presented as the median together with the interquartile range. Categorical data were presented as percentages. The Kolmogorov–Smirnov test was utilized to assess the normal distribution of continuous variables. And if the variables followed a normal distribution, they were analyzed with either a t-test or an ANOVA. If the distribution was non-normal, statistical analysis was performed with the Mann–Whitney U-test or Kruskal-Wallis test. The Kaplan–Meier (K-M) survival analysis was utilized to evaluate the event rate in groupings categorized by varying SII levels, and any discrepancies were examined through log-rank analysis. Binary logistic regression was employed to assess variables that influence the likelihood of all-cause mortality. Employing Cox’s proportional hazards regression models, we ascertained the hazard ratio (HR), along with its 95% confidence interval (CI), relating to the SII and the endpoints, incorporating adjustments in select models. Variables having a *p*-value below 0.05 in univariate analysis were deemed confounders. Clinically pertinent factors along with those linked to prognosis were incorporated into the multivariate analysis for model: model 1: unadjusted; model 2: adjusted for age, sex, and BMI; model 3: adjusted for age, sex, BMI, sepsis, dementia, BUN, PT-INR, calcium, hemoglobin, and mechanical ventilation. For improved clarity in the representation of the hazard ratio (HR), the SII value was divided by 1,000.Further, employing restricted cubic splines (RCS) characterized by four knots, we investigated the non-linear correlation between the initial SII score and all-cause mortality rates within 30 days and 1 year after hospital admission. The assessment and identification of the SII’s cutoff point are carried out through an analysis of the receiver operating characteristic (ROC) curves. Models incorporated the SII either as a continuous metric or an ordinal variable, with the lowest quartile of the SII serving as the baseline category. Trend *p*-values were determined using quartile categories. Separate analyses were performed according to gender, age (above 80 and 80 years or less), BMI (over 30 and 30 kg/m^2^ or less), sepsis, dementia, and osteoporosis, in order to assess the uniformity of SII’s predictive strength for principal outcomes. To investigate the interaction effects between SII and the stratification factors, likelihood ratio tests were applied. A statistically noteworthy threshold was established with a two-tailed *p*-value below 0.05. In addition, we applied the Spearman correlation analysis to examine the relationship between different severity of illness scores and SII. Then we apply receiver operating characteristic (ROC) analysis to assess the predictive capacity of different severity of illness scores for 30-day and 1-year mortality in critically ill elderly patients with hip fracture. What’s more, the data analysis was carried out utilizing R software, specifically version 4.0.2, along with SPSS 25.0 (IBM SPSS Statistics, Armonk, NY, United States).

## 3 Results

This study encompassed a number of 991 geriatric patients with hip fractures, whose median age stood at 81.63 years (interquartile range: 75–89), among which 357 (36.02%) were males. The mean SII index across all participants was calculated to be 2115.26 (interquartile range: 655.11–2379.63). The recorded mortality rates were 19.68% within 30 days post-admission and 33.40% at the one-year mark (Table 1).

**Table 1:** Features and results of participants grouped by SII.

Variable	Total (*n* = 991)	Q1 (*n* = 248)	Q2 (*n* = 248)	Q3 (*n* = 248)	Q4 (*n* = 247)	X^2/F^	*p*-value
Age (year)	81.63 (75–89)	80.6 (73.5–88)	82.4 (77.5–89)	82.1 (74–90.5)	81.4 (74–88)	2.289	0.106
Sex: male	357 (36.02)	87 (35.08)	86 (34.68)	95 (38.31)	89 (36.03)	0.283	0.837
BMI	25.89 (21.51–28.98)	26.39 (22.54–29.99)	25.21 (21.25–28.13)	26.28 (21.90–29.27)	25.66 (20.82–28.72)	2.096	0.049
Temperature (°C)	36.79 (36.56–37.02)	36.81 (36.55–37.02)	36.77 (36.52–37.00)	36.79 (36.58–37.03)	36.79 (36.58–37.01)	0.264	0.822
Heart rate (beats/min)	84.66 (73.92–83.69)	83.45 (72.56–93.52)	83.00 (72.54–93.45)	85.50 (74.63–93.5)	86.69 (75.58–96.52)	3.393	0.019
Respiratory rate (breaths/min)	19.45 (16.72–21.71)	19.18 (16.67–21.45)	18.89 (16.39–20.98)	19.45 (16.60–22.17)	20.30 (17.35–22.55)	6.701	0.001
SBP (mmHg)	118.28 (105.58–129.40)	98.85 (95.54–98.41)	96.89 (95.70–98.35)	96.79 (95.52–98.30)	96.45 (95.19–97.74)	0.658	0.627
DBP (mmHg)	59.38 (51.69–66.08)	60.40 (52.67–67.55)	58.47 (51.42–64.69)	59.31 (52.03–65.83)	59.34 (50.61–66.16)	1.346	0.239
MBP (mmHg)	75.12 (67.52–81.62)	76.15 (68.54–83.51)	74.63 (67.86–80.45)	74.61 (66.87–81.37)	75.09 (66.56–81.80)	1.111	0.416
SpO2 (%)	96.74 (95.50–98.24)	96.85 (95.54–98.41)	96.89 (95.70–98.35)	96.79 (95.51–98.30)	96.45 (95.19–97.74)	2.569	0.069
Commorbidities							
Diabetes mellitus	229 (23.1)	66 (26.61)	54 (21.77)	64 (25.81)	45 (18.22)	6.708	0.082
Rheumatic Disease	77 (7.77)	19 (7.66)	20 (8.06)	22 (8.87)	16 (6.48)	0.106	0.776
COPD	131 (13.22)	33 (13.31)	21 (8.47)	34 (13.71)	43 (17.41)	2.919	0.033
PE	59 (5.95)	14 (5.65)	13 (5.24)	17 (6.85)	15 (6.07)	0.210	0.889
DVT	79 (7.97)	18 (7.26)	16 (6.45)	28 (11.29)	17 (6.88)	1.694	0.166
Dementia	71(7.16)	18 (7.26)	15 (6.05)	19 (7.66)	19 (7.69)	0.220	0.882
Coronary heart disease	324 (32.69)	81 (32.66)	83 (3.35)	78 (31.45)	82 (33.20)	0.090	0.966
Osteoporosis	205 (20.69)	52 (20.97)	54 (21.77)	51 (20.56)	48 (19.43)	0.143	0.934
Sepsis	61 (6.16)	11 (4.44)	9 (3.63)	15 (6.05)	26 (10.53)	4.095	0.007
CKD	220 (22.20)	56 (22.58)	54 (21.77)	55 (22.18)	55 (22.27)	0.016	0.997
Pneumonia	41 (4.14)	6 (2.42)	11 (4.44)	10 (4.03)	14 (5.67)	1.122	0.338
Cerebral infarction	128 (12.92)	35 (14.11)	27 (10.89)	32 (12.90)	34 (13.77)	0.459	0.710
Hypertension	185 (18.67)	40 (16.13)	57 (22.98)	43 (17.34)	45 (18.22)	1.473	0.220
Laboratory tests							
Aniongap (mEq/L)	14.8 (12–17)	14.24 (12–16)	14.54 (12–17)	15.26 (13–17)	15.25 (12.5–17)	4.379	0.007
CK-MB (ng/ml)	9.9 (3.0–13.6)	11.41 (3.5–15.68)	9.79 (3–14.38)	9.55 (3–12.06)	8.90 (3–12.12)	2.495	0.117
BUN (mg/dL)	30.0 (17–35.5)	29.77 (18–34.5)	27.84 (15.5–30.27)	30.63 (16–38.5)	31.94 (18–42)	1.743	0.030
ALP (U/L)	100.6 (61.3–124.0)	98.37 (58.74–121.17)	101.16 (63.50–130.72)	99.01 (60.18–117.35)	104.02 (66–127)	0.494	0.220
Bicarbonate (mmol/L)	24.9 (22–28)	24.53 (22–28)	25.15 (22.5–28)	25.03 (23–27)	24.86 (22–28)	1.032	0.526
Totalbilirubin (mg/dL)	1.0 (0.4–1.2)	1.09 (0.4–1.3)	1.03 (0.45–1.29)	1.01 (0.4–1.27)	0.95 (0.35–1.1)	0.946	0.025
Sodium (mmol/L)	137.8 (135–141)	138.48 (136–141)	137.42 (135.25–140)	137.82 (134.75–141)	137.68 (135–141)	1.770	0.489
Chloride (mmol/L)	103.7 (100–107)	104.73 (101–108)	103.07 (100–107)	103.52 (100–107)	103.39 (100–107)	3.274	0.036
Calcium (mmol/L)	5.5 (1.1–8.5)	5.42 (1.12–8.5)	5.81 (1.16–8.7)	5.63 (1.13–8.6)	4.95 (1.11–8.3)	2.571	0.005
Potassium(mmol/L)	4.2 (3.6–4.6)	4.29 (3.8–4.68)	4.23 (3.73–4.6)	4.32 (3.8–4.7)	4.15 (3.6–4.5)	2.377	0.175
PT (s)	16.3 (12.3–16.3)	16.16 (12.3–16.3)	15.69 (12.2–16.3)	16.99 (12.5–16.65)	16.38 (12.4–16.3)	1.063	0.765
PTT (s)	37.0 (27.8–38.1)	36.31 (27.9–38.43)	36.24 (27.8–37.85)	39.53 (27.7–39.35)	36.05 (27.7–37.5)	2.035	0.963
PT-INR	1.47 (1.1–1.5)	1.48 (1.1–1.5)	1.46 (1.1–1.5)	1.54 (1.1–1.6)	1.44 (1.1–1.45)	0.762	0.224
Creatinine(mg/dL)	1.0 (0.6–1)	1.06 (0.6–1.02)	1.02 (0.6–1)	1.01 (0.6–1.02)	1.04 (0.6–1)	0.140	0.622
WBC counts (10^9^/L)	11.8 (7.4–14.1)	9.24 (5.75–10.6)	10.20 (6.85–12.2)	11.84 (8.35–13.85)	15.93 (10.6–18.7)	53.606	<0.001
Platelet counts (10^9^/L)	209.8 (145–260)	143.48 (92.5–187)	201.64 (150.5–243.75)	226.81 (162.25–274)	267.53 (192–325)	96.981	<0.001
Lymphocyte counts (10^9^/L)	1.4 (0.8–1.6)	2.11 (1.12–2.11)	1.43 (0.97–1.75)	1.19 (0.83–1.42)	0.81 (0.51–1.01)	26.106	<0.001
Neutrophil counts (10^9^/L)	8.8 (5.0–10.9)	4.67 (3.09–5.81)	6.82 (4.89–8.55)	9.46 (6.92–11.07)	14.16 (9.27–16.91)	196.272	<0.001
Monocyte count (10^9^/L)	0.6 (0.3–0.7)	0.48 (0.32–0.58)	0.55 (0.34–0.64)	0.59 (0.37–0.72)	0.69 (0.35–0.82)	7.517	<0.001
Hemoglobin (g/dL)	10.1 (8.6–11.4)	10.03 (8.55–11.25)	10.19 (8.75–11.6)	10.02 (8.5–11.38)	10.19 (8.6–11.5)	0.623	0.733
Hematocrit (%)	31.2(26.9–35.2)	31.00 (26.95–34.8)	31.23 (26.85–35.53)	31.02 (26.3–35.05)	31.43 (27.1–35.4)	0.308	0.873
Heparin	635 (64.08)	144 (58.06)	152 (61.29)	168 (67.74)	171 (68.95)	3.025	0.029
Mechanical ventilation	173 (17.46)	41 (16.53)	36 (14.52)	48 (19.35)	48 (19.43)	0.974	0.403
SII	2115.26 (655.11–2379.63)	386.28 (247.90–529.49)	931.46 (783.58–1084.92)	1734.64 (1459.44–2019.21)	5422.02 (3006.13–6,358)	218	<0.001
Severity of illness scores							
SOFA	1.59 (1–2)	1.40 (1–1.77)	1.46 (1–2)	1.58 (1–2)	1.74 (1–2)	1.537	0.039
SAPS II	41.46 (33–47)	41.08 (32.5–48)	40.71 (33–46)	42.16 (34–48)	41.89 (33–48)	0.784	0.527
LODS	4.93 (3–6)	4.76 (3–6)	4.68 (3–6)	5.29 (3–7)	4.99 (3–6)	2.055	0.072
OASIS	34.01 (28–39)	33.33 (26–39)	33.46 (28–38)	34.38 (29–39)	34.87 (29–40)	1.992	0.122
Events							
30-day mortality	195 (19.68)	25 (10.08)	45 (18.15)	52 (20.97)	73 (29.55)	10.386	<0.001
1-year mortality	331 (33.40)	66 (26.61)	75 (30.24)	87 (30.08)	103 (41.70)	4.787	0.003

SII, systemic immune-inflammation index; BMI, body mass index; SBP, systolic blood pressure; DBP, diastolic blood pressure; MBP, mean blood pressure; SpO2, pulse oximetry-derived oxygen saturation; COPD, chronic obstructive pulmonary disease; PE, pulmonary embolism; DVT, deep venous thrombosis; CKD, chronic kidney disease; CK-MB, Creatine Kinase Isoenzyme-MB;BUN, blood urea nitrogen; ALP, alkaline phosphatase; PT, prothrombin time; PTT, partial thromboplastin time; WBC, white blood cell; SOFA, sequential organ failure assessment; SAPS II, simplifed acute physiological score II; LODS, the logistic organ dysfunction system; OASIS, oxford acute severity of illness score.

### 3.1 Baseline characteristics

Fundamental attributes of severely ill hip fracture patients segmented according to their SII quartiles are presented in Table 1.Participants were categorized into four distinct groups according to their SII levels at the time of hospital admission.[quartile Q1: 73.04–655.11; Q2: 655.11–1231.57; Q3: 1231.57–2379.63; Q4: 2379.63–12685.89]. The median SII value for each quartile was 386.28 (IQR: 247.90–529.49), 931.46 (IQR: 783.58–1084.92), 1734.64 (IQR: 1459.44–2019.21), and 5422.02 (IQR: 3006.13–6,358), respectively. Patients in the highest quartile of SII exhibited elevated heart rate and respiratory rate, along with a higher incidence of COPD and sepsis. Their white blood cell (WBC) counts, anion gap, and blood urea nitrogen (BUN) concentrations were heightened, while BMI, total bilirubin, chloride, and calcium levels exhibited a decline. Additionally, individuals in their group exhibited a greater severity of illness upon admission when compared with those in the lower quartile. The ranking in the upper quartile of the SII exhibited a notably increased mortality rate after 1 year when contrasted with individuals in the lower quartile (26.61% vs. 30.24% vs. 30.08% vs. 41.70%, *p* = 0.003). Similar results were observed in individuals with a 30-day mortality rate (10.08% vs. 18.15% vs. 20.97% vs. 29.55%, *p* < 0.001). Table 2 shows the differing initial characteristics of individuals who survived and those who did not survive 30 days after being admitted. Follow-up at 30 days after admission showed a mortality rate of 19.68%, with 195 patients dying and 796 patients surviving. No data loss occurred during the 30-day follow-up. Individuals in the non-survivor group exhibited a greater likelihood of being male, a greater incidence of dementia and sepsis, elevated levels of PT-INR, BUN, PT, and WBC, decreased levels of calcium and chloride, increased usage of mechanical ventilation, and higher severity of disease scores. The levels of SII were significantly higher in the group of patients who did not survive as compared to those who did (3389.64 vs. 1803.07, *p* < 0.001). Table 3 shows the differing initial characteristics of individuals who survived and those who did not survive 1 year after being admitted. Follow-up at 1 year after admission showed a mortality rate of 33.40%, with 331 patients dying and 660 patients surviving. No data loss occurred during the 1-year follow-up. The characteristics of patients in the non-survivor group 1 year after admission were similar to those at 1 month after admission, but we observed that this group tended to have a higher prevalence of chronic kidney disease, coronary artery disease, and osteoporosis, higher creatinine, and lower total bilirubin compared with survivors. In the group of patients who did not survive, the level of SII was significantly higher than in the group of patients who did survive (2646.79 vs. 1848.69, *p* < 0.001).

**Table 2:** Baseline characteristics of the survivors and Non-survivors groups at 30 days following hospital admission.

Variable	Total (*n* = 991)	Survivor (*n* = 796)	Non-survivor (*n* = 195)	*p*-value
Ages (year)	81.63 (75–89)	81.09 (74–88)	83.85 (79–91)	<0.001
Sex: male	357 (36.02)	275 (34.55)	82 (42.05)	0.050
BMI	25.89 (21.51–28.98)	26.1 (21.7–29.1)	25.0 (15.6–43.4)	0.018
Temperature (°C)	36.79 (36.56–37.02)	36.8 (36.6–37.0)	36.7 (36.5–37.0)	0.153
Heart rate (beats/min)	84.66 (73.92–83.69)	84.4 (73.7–93.8)	85.7 (75.6–95.4)	0.119
Respiratory rate (breaths/min)	19.45 (16.72–21.71)	19.3 (16.7–21.5)	20.0 (17.1–22.3)	0.065
SBP (mmHg)	118.28 (105.58–129.40)	119.0 (106.3–130.2)	115.4 (103.9–126.1)	0.006
DBP (mmHg)	59.38 (51.69–66.08)	59.6 (51.9–66.6)	58.4 (50.8–64.9)	0.081
MBP (mmHg)	75.12 (67.52–81.62)	75.5 (68.0–82.2)	73.7 (65.9–80.2)	0.021
SpO2 (%)	96.74 (95.50–98.24)	96.8 (95.6–98.2)	96.6 (95.1–98.2)	0.464
Commorbidities				
Diabetes mellitus	229 (23.1)	187 (23.5)	42 (21.54)	0.317
Rheumatic Disease	77 (7.77)	66 (8.29)	11 (5.64)	0.136
COPD	131 (13.22)	109 (13.69)	22 (11.28)	0.373
PE	59 (5.95)	45 (6.03)	14 (7.18)	0.420
DVT	79 (7.97)	63 (7.91)	16 (8.21)	0.893
Dementia	71 (7.16)	50 (6.28)	21 (10.77)	0.029
Coronary heart disease	324 (32.69)	252 (31.66)	72 (36.92)	0.160
Osteoporosis	205 (20.69)	172 (21.61)	33 (16.92)	0.148
Sepsis	61 (6.16)	33 (4.15)	28 (14.36)	<0.001
CKD	220 (22.20)	169 (21.23)	51 (26.15)	0.138
Pneumonia	41 (4.14)	31 (3.89)	10 (5.13)	0.438
Cerebral infarction	128 (12.92)	96 (12.06)	32 (16.41)	0.105
Hypertension	185 (18.67)	149 (18.72)	36 (18.46)	0.934
Laboratory tests				
Anion gap (mEq/L)	14.8 (12–17)	14.73 (12–16.5)	15.19 (12–17)	0.261
CK-MB(ng/ml)	9.9 (3.0–13.6)	9.90 (3–13.3)	9.98 (3.10–14.55)	0.490
BUN (mg/dL)	30.0 (17–35.5)	28.95 (16–33.75)	34.51 (20–43)	<0.001
ALP(U/L)	100.6 (61.3–124.0)	98.76 (60–121.33)	108.28 (67–134)	0.060
Bicarbonate(mmol/L)	24.9 (22–28)	24.95 (22.25–28)	24.66 (22–28)	0.470
Total bilirubin (mg/dL)	1.0 (0.4–1.2)	1.01 (0.4–1.2)	1.08 (0.4–1.28)	0.661
Sodium (mmol/L)	137.8 (135–141)	137.79 (135–141)	138.10 (135–141)	0.647
Chloride (mmol/L)	103.7 (100–107)	103.50 (100–107)	104.38 (101–108)	0.110
Calcium (mmol/L)	5.5 (1.1–8.5)	5.66 (1.14–8.55)	4.61 (1.1–8.25)	0.001
Potassium (mmol/L)	4.2 (3.6–4.6)	4.24 (3.75–4.6)	4.29 (3.7–4.7)	0.106
PT(s)	16.3 (12.3–16.3)	16.05 (12.3–16.3)	17.35 (12.7–17.4)	0.007
PTT(s)	37.0 (27.8–38.1)	36.91 (27.8–37.9)	37.54 (27.9–41.1)	0.459
PT-INR	1.47 (1.1–1.5)	1.45 (1.1–1.5)	1.60 (1.1–1.7)	0.043
Creatinine (mg/dL)	1.0 (0.6–1)	1.01 (0.6–1)	1.12 (0.6–1.2)	0.371
WBC counts (10^9^/L)	11.8 (7.4–14.1)	11.40 (7.3–13.78)	13.42 (8.1–16.8)	0.001
Platelet counts (10^9^/L)	209.8 (145–260)	208.42 (145–259.75)	215.47 (143–271)	0.566
Lymphocyte counts (10^9^/L)	1.4 (0.8–1.6)	1.44 (0.84–1.66)	1.16 (0.62–1.38)	<0.001
Neutrophil counts (10^9^/L)	8.8 (5.0–10.9)	8.31 (4.81–10.49)	10.65 (6.16–13.50)	<0.001
Monocyte counts (10^9^/L)	0.6 (0.3–0.7)	0.57 (0.34–0.66)	0.62 (0.37–0.76)	0.014
Hemoglobin (g/dL)	10.1 (8.6–11.4)	10.15 (8.6–11.5)	9.53 (8.5–11.2)	0.134
Hematocrit (%)	31.2 (26.9–35.2)	31.2 (26.7–35.3)	31.0 (27.3–34.7)	0.612
Heparin	635 (64.08)	508 (63.82)	127 (65.13)	0.773
Mechanical ventilation	173 (17.46)	128 (16.08)	45 (23.08)	0.021
SII	2115.26 (655.11–2379.63)	1803.07 (595.67–2178.48)	3389.64 (927.3–3,652)	<0.001
Severity of illness scores				
SOFA	1.59 (1–2)	1.51 (1–1.53)	1.93 (1–3)	0.009
SAPS II	41.46 (33–47)	40.28 (33–46)	46.27 (37–54)	<0.001
LODS	4.93 (3–6)	4.67 (3–6)	6.01 (4–8)	<0.001
OASIS	34.01 (28–39)	33.34 (28–38)	36.75 (31–43)	<0.001

SII, systemic immune-inflammation index; BMI, body mass index; SBP, systolic blood pressure; DBP, diastolic blood pressure; MBP, mean blood pressure; SpO2, pulse oximetry-derived oxygen saturation; COPD, chronic obstructive pulmonary disease; PE, pulmonary embolism; DVT, deep venous thrombosis; CKD, chronic kidney disease; CK-MB, Creatine Kinase Isoenzyme-MB;BUN, blood urea nitrogen; ALP, alkaline phosphatase; PT, prothrombin time; PTT, partial thromboplastin time; WBC, white blood cell; SOFA, sequential organ failure assessment; SAPS II, simplifed acute physiological score II; LODS, the logistic organ dysfunction system; OASIS, oxford acute severity of illness score.

**Table 3:** Baseline characteristics of the survivors and Non-survivors groups at 1-year following hospital admission.

Variable	Total (*n* = 991)	survivor (*n* = 660)	Non-survivor (*n* = 331)	*p*-value
Ages (year)	81.63 (75–89)	80.49 (74–87)	83.92 (79–91)	<0.001
Sex: male	357 (36.02)	221 (33.48)	136 (41.09)	0.019
BMI	25.89 (21.51–28.98)	26.47 (22.20–29.32)	24.72 (20.24–28.08)	<0.001
Temperature (°C)	36.79 (36.56–37.02)	36.82 (36.58–37.05)	36.72 (36.49–36.97)	0.001
Heart rate (beats/min)	84.66 (73.92–83.69)	84.51 (73.77–93.72)	84.95 (74.21–94.75)	0.658
Respiratory rate (breaths/min)	19.45 (16.72–21.71)	19.27 (16.76–21.48)	19.82 (16.65–22.48)	0.154
SBP (mmHg)	118.28 (105.58–129.40)	119.53 (107.21–130.72)	115.79 (103.16–126.96)	0.001
DBP (mmHg)	59.38 (51.69–66.08)	60.10 (52.41–66.76)	57.95 (50.56–64.88)	0.004
MBP (mmHg)	75.12 (67.52–81.62)	76.02 (68.48–82.44)	73.34 (65.17–79.63)	0.001
SpO2 (%)	96.74 (95.50–98.24)	96.82 (95.61–98.24)	96.60 (95.19–98.24)	0.392
Commorbidities				
Diabetes mellitus	229 (23.1)	148 (22.42)	81 (24.47)	0.260
Rheumatic Disease	77 (7.77)	57 (8.64)	20 (6.04)	0.093
COPD	131 (13.22)	87 (13.18)	44 (13.29)	0.961
PE	59 (5.95)	31 (4.70)	28 (8.46)	0.018
DVT	79 (7.97)	45 (6.82)	34 (10.27)	0.058
Dementia	71 (7.16)	26 (3.94)	45 (13.60)	<0.001
Coronary heart disease	324 (32.69)	201 (30.45)	123 (37.16)	0.034
Osteoporosis	205 (20.69)	152 (23.03)	53 (16.01)	0.010
Sepsis	61 (6.16)	23 (3.48)	38 (10.57)	<0.001
CKD	220 (22.20)	124 (18.79)	96 (29.00)	<0.001
Pneumonia	41 (4.14)	22 (3.33)	19 (5.74)	0.073
Cerebral infarction	128 (12.92)	81 (12.27)	47 (14.20)	0.394
Hypertension	185 (18.67)	131 (19.85)	54 (16.31)	0.178
Laboratory tests				
Anion gap (mEq/L)	14.8 (12–17)	14.63 (12–16.5)	15.19 (12.5–17)	0.081
CK-MB (ng/ml)	9.9 (3.0–13.6)	10.29 (1.02–14.58)	9.18 (3.52–12.25)	0.953
BUN (mg/dL)	30.0 (17–35.5)	27.59 (15.75–32)	34.94 (21–43)	<0.001
ALP (U/L)	100.6 (61.3–124.0)	98.78 (61.13–121.16)	104.34 (62–130.5)	0.133
Bicarbonate (mmol/L)	24.9 (22–28)	25.01 (22.5–28)	24.65 (22–28)	0.459
Total bilirubin (mg/dL)	1.0 (0.4–1.2)	1.03 (0.4–1.3)	0.99 (0.4–1.1)	0.029
Sodium (mmol/L)	137.8 (135–141)	137.54 (135–141)	138.46 (136–141)	0.041
Chloride (mmol/L)	103.7 (100–107)	103.44 (100–107)	104.15(100–108)	0.189
Calcium (mmol/L)	5.5 (1.1–8.5)	5.73 (1.14–8.58)	4.90 (1.12–8.4)	0.010
Potassium (mmol/L)	4.2 (3.6–4.6)	4.25 (3.78–4.6)	4.25 (3.7–4.65)	0.751
PT(s)	16.3 (12.3–16.3)	15.82 (12.2–16.3)	17.27 (12.7–17.4)	<0.001
PTT(s)	37.0 (27.8–38.1)	36.62 (27.5–36.8)	37.85 (28.1–40.9)	0.063
PT-INR	1.47 (1.1–1.5)	1.42 (1.1–1.5)	1.59 (1.1–1.6)	0.012
Creatinine (mg/dL)	1.0 (0.6–1)	0.98 (0.6–1)	1.15 (0.6–1.2)	0.002
WBC counts (10^9^/L)	11.8 (7.4–14.1)	11.50 (7.4–13.7)	12.39 (7.4–15.3)	0.047
Platelet counts (10^9^/L)	209.8 (145–260)	210.98 (149.25–260)	207.48 (138–262)	0.180
Lymphocyte counts (10^9^/L)	1.4 (0.8–1.6)	1.42 (0.84–1.68)	1.32 (0.69–1.40)	<0.001
Neutrophil counts (10^9^/L)	8.8 (5.0–10.9)	8.37 (4.88–10.27)	9.58 (5.31–11.97)	0.002
Monocyte counts (10^9^/L)	0.6 (0.3–0.7)	0.57 (0.34–0.66)	0.58 (0.34–0.7)	0.455
Hemoglobin (g/dL)	10.1 (8.6–11.4)	10.19 (8.65–11.5)	9.94 (8.5–11.25)	0.059
Hematocrit (%)	31.2 (26.9–35.2)	31.40 (26.95–35.6)	30.71 (26.5–34.6)	0.088
Heparin	635 (64.08)	421 (63.79)	214 (64.65)	0.789
Mechanical ventilation	173 (17.46)	99 (15.00)	74 (22.36)	0.004
SII	2115.26 (655.11–2379.63)	1848.69 (605.52–2180.83)	2646.79 (830.25–2867.27)	<0.001
Severity of illness scores				
SOFA	1.59 (1–2)	1.57 (1–1.53)	1.65 (1–2)	0.880
SAPS II	41.46 (33–47)	39.18 (32–44)	46.01 (37–52)	<0.001
LODS	4.93 (3–6)	4.41 (2–6)	5.97 (4–8)	<0.001
OASIS	34.01 (28–39)	32.82 (27–37.5)	36.39 (31–42)	<0.001

SII, systemic immune-inflammation index; BMI, body mass index; SBP, systolic blood pressure; DBP, diastolic blood pressure; MBP, mean blood pressure; SpO2, pulse oximetry-derived oxygen saturation; COPD, chronic obstructive pulmonary disease; PE, pulmonary embolism; DVT, deep venous thrombosis; CKD, chronic kidney disease; CK-MB, Creatine Kinase Isoenzyme-MB;BUN, blood urea nitrogen; ALP, alkaline phosphatase; PT, prothrombin time; PTT, partial thromboplastin time; WBC, white blood cell; SOFA, sequential organ failure assessment; SAPS II, simplifed acute physiological score II; LODS, the logistic organ dysfunction system; OASIS, oxford acute severity of illness score.

### 3.2 Primary outcomes

**Figure 2:**
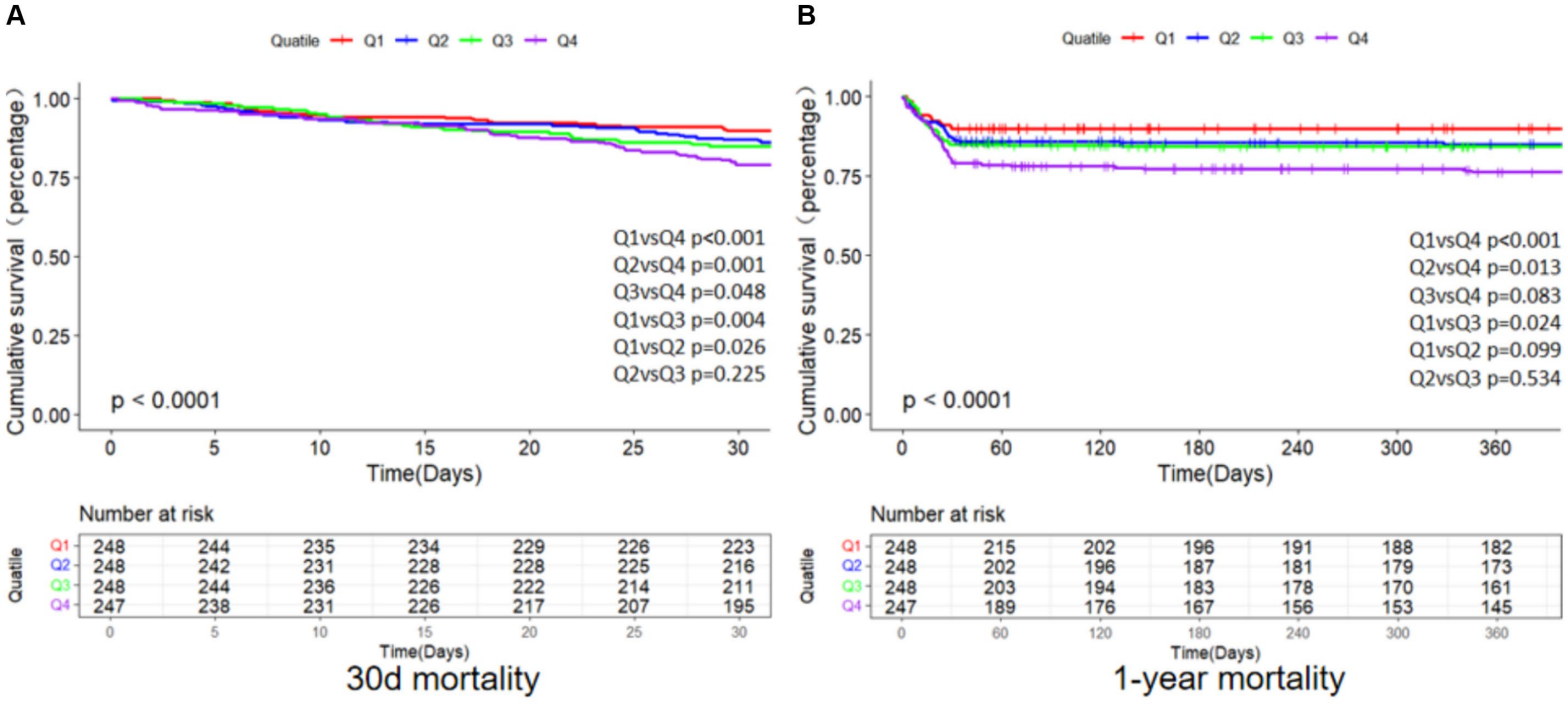
K-M survival analysis curves for all-cause mortality of 30 days **(A)** and 1 year **(B)**. Footnote SII quartiles: Q1 (73.04–655.11), Q2 (655.11–1231.57), Q3 (1231.57–2379.63), Q4 (2379.63–12685.89).

As depicted in Figure 2, the primary endpoints’ incidence across distinct clusters, demarcated by SII quartiles, was investigated through the employment of the K-M survival analysis curves. A notable disparity was observed in the mortality rates when comparing the brief (30-day) and extended (1-year) intervals, revealing a statistically noteworthy distinction (log-rank *p*-value less than 0.001).Furthermore, patients with an elevated SII faced an increased likelihood of mortality between 30 days and 1 year following admission. The SII’s clinical effectiveness was evaluated through ROC analysis. The AUC values for SII were not good enough: 30-day death AUC was 0.629 (*p* < 0.001) and 1-year death AUC was 0.576 (*p* < 0.001) (Supplementary Figure S1). Supplementary Table S2 displays the findings from binary logistic regression analysis regarding the likelihood of all-cause mortality among geriatric patients with hip fractures. Independent variables for logistic regression encompassed those factors that showed significance in univariate analysis with a threshold of *p* < 0.05, along with propositions from clinicians and insights drawn from clinical practice. The results found that sex, age, sepsis, calcium, hemoglobin, and SII were significant predictors.

**Figure 3:**
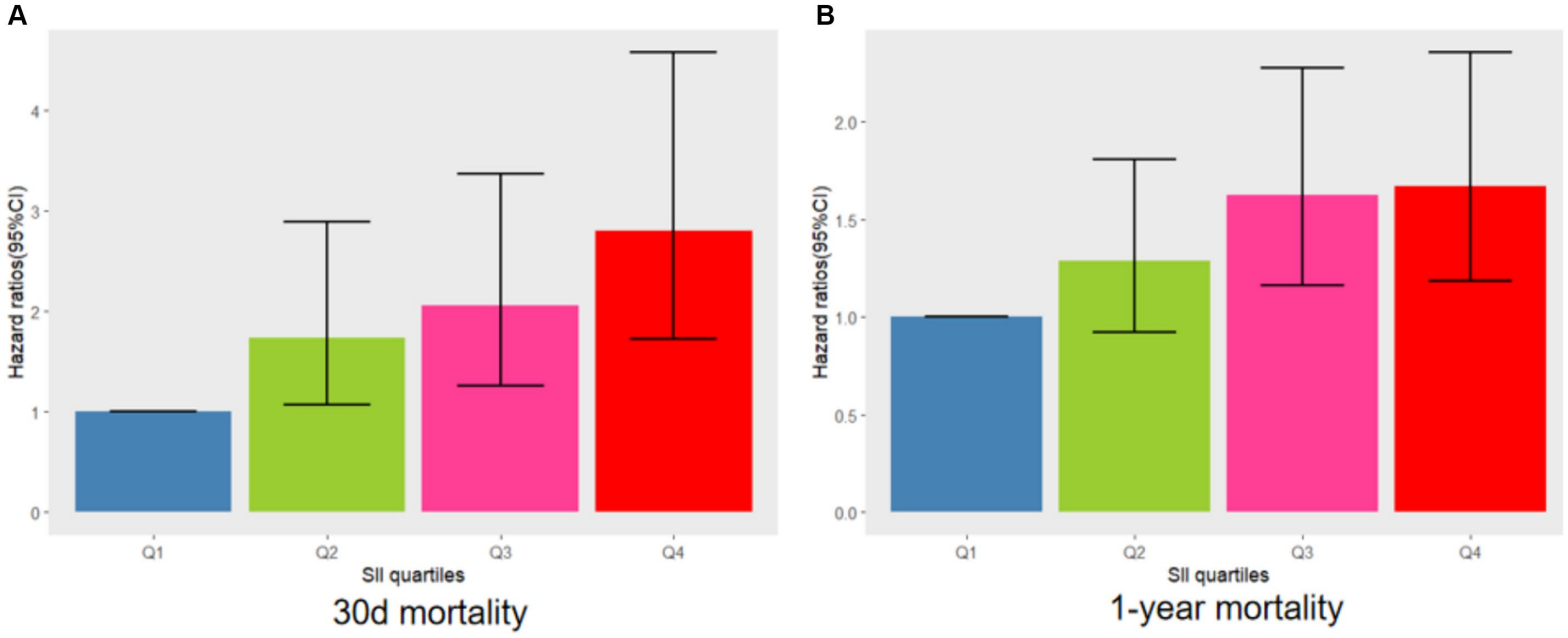
Adjusted hazard ratios with 95% confidence intervals (95% CIs) for 30 days **(A)** and 1 year **(B)** mortality based on SII quartiles after controlling for age, BMI, sex, sepsis, dementia, BUN, PT-INR, calcium, hemoglobin, and mechanical ventilation. CI, confdence interval; SII, systemic immune-inflammation index.

**Figure 4:**
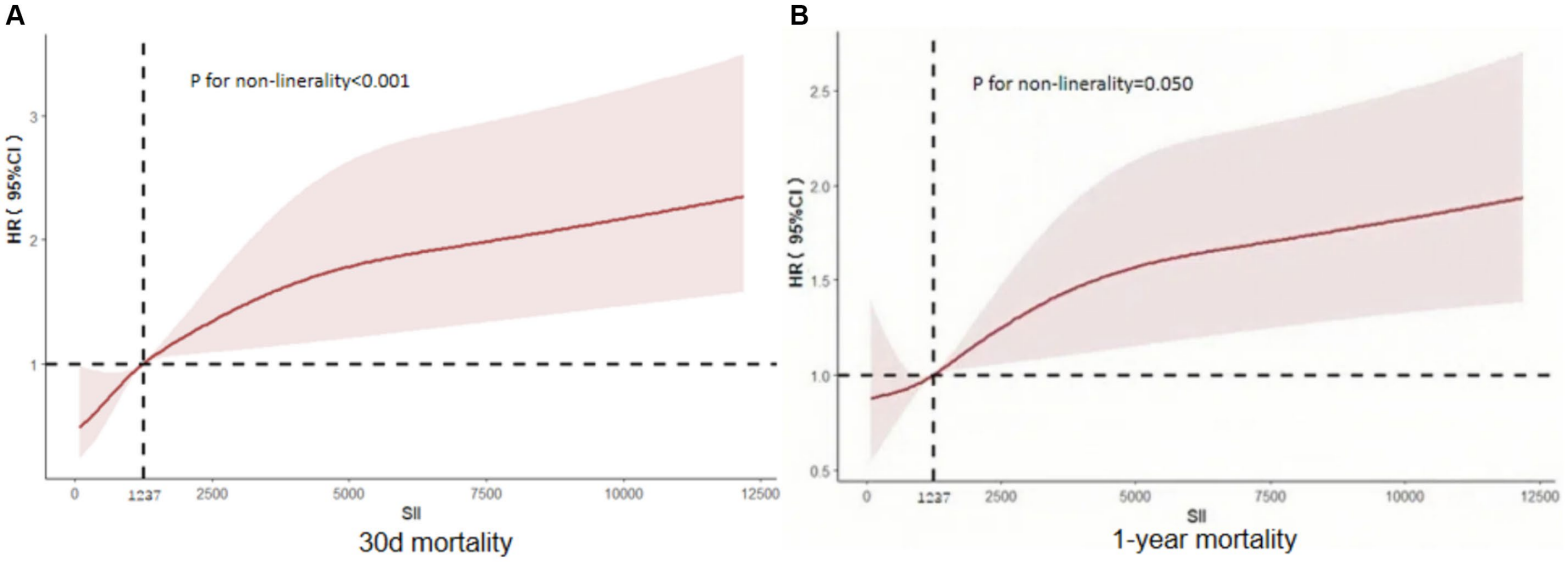
Restricted cubic spline curve depicting the hazard ratio of 30 days **(A)** and 1 year **(B)** for the SII. CI, confdence interval; SII, systemic immune-inflammation index.

The relationship between SII and 30-day mortality was investigated using a Cox proportional hazards regression analysis. Analysis revealed that the SII consistently emerged as a significant risk factor across all models when examined as a continuous variable: model 1 [HR, 1.073 (95% CI 1.019–1.090) *p* = 0.002], model 2 [HR, 1.062 (1.043–1.08) *p* < 0.001], and model 3 [HR, 1.065 (1.044–1.087) *p* < 0.001]. In the context where SII was categorized, individuals falling into the highest quartile of SII demonstrated a notably heightened likelihood of 30-day mortality across three distinct Cox proportional hazards models: model 1 [HR, 2.649 (95% CI 1.30–5.399) *p* = 0.007], model 2 [HR, 2.635 (95% CI 1.658–4.188) *p* < 0.001], and model 3 [HR, 2.806 (95% CI 1.721–4.577) *p* < 0.001], compared to those in the lowest quartile. This risk tended to increase with higher SII values (Table 4; Figure 3A). The multivariate Cox proportional hazard analysis indicated similar outcomes for the SII and 1-year mortality (Table 4; Figure 3B). What’s more,the study utilized a confined cubic splines regression analysis to exhibit that both the 30-day and 1-year mortality rates escalated non-linearly with escalating SII (with *p* values for non-linearity equating to 0.050 and less than 0.001, respectively) (Figures 4A,B).

**Table 4:** Cox proportional hazard ratios (HR) for all-cause mortality.

Categories	Model 1			Model 2			Model 3		
	HR (95%CI)	*p*-value	P for trend	HR (95%CI)	*p*-value	P for trend	HR (95%CI)	*p*-value	P for trend
30-day mortality									
Continuous variable per unit	1.053 (1.019–1.090)	0.002		1.062 (1.043–1.081)	<0.001		1.065 (1.044–1.087)	<0.001	
Quartile			0.037			0.019			0.01
Q1 (*N* = 248)	Ref								
Q2 (*N* = 248)	1.85 (1.072–3.192)	0.027		1.630 (0.992–2.676)	0.054		1.735 (1.062–2.895)	0.028	
Q3 (*N* = 248)	2.21 (1.22–4.003)	0.009		2.006 (1.243–3.238)	0.004		2.061 (1.261–3.368)	0.004	
Q4 (*N* = 247)	2.649 (1.3–5.399)	0.007		2.635 (1.658–4.188)	<0.001		2.806 (1.721–4.577)	<0.001	
1-year mortality									
Continuous variable per unit	1.058 (1.028–1.089)	<0.001		1.049 (1.028–1.070)	<0.001		1.051 (1.029–1.074)	<0.001	
Quartile			0.049			0.026			0.013
Q1 (*N* = 248)	Ref								
Q2 (*N* = 248)	1.176 (0.844–1.640)	0.338		1.227 (0.877–1.718)	0.232		1.290 (0.919–1.809)	0.141	
Q3 (*N* = 248)	1.405 (1.018–1.938)	0.039		1.493 (1.072–2.079)	0.018		1.627 (1.161–2.279)	0.005	
Q4 (*N* = 247)	1.582 (1.153–2.172)	0.005		1.548 (1.099–2.181)	0.012		1.670 (1.184–2.356)	0.003	

Model 1: unadjusted Model 2: adjusted for age, sex, BMI Model 3: adjusted for age, sex, BMI, sepsis, dementia, BUN, PT-INR, calcium, hemoglobin, and mechanical ventilation.

**Figure 5:**
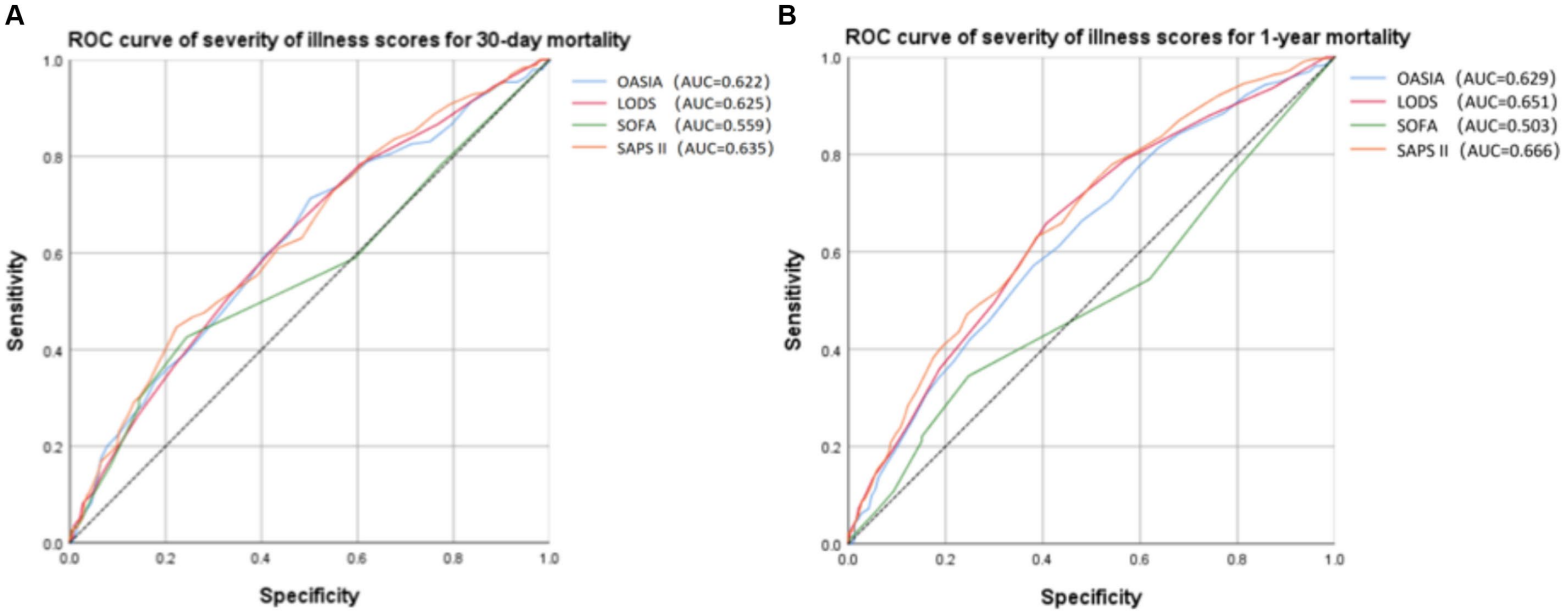
Receiver operating characteristic (ROC) curve of severity of illness scores for 30-day **(A)** and 1-year **(B)** mortality.

Receiver operating characteristic (ROC) analysis was conducted to assess the predictive capacity of different severity of illness scores for 30-day and 1-year mortality in critically ill elderly patients with hip fracture (Figure 5). Based on the AUC values (Figures 5A,B, Supplementary Tables S3, S4), the SAPS II score for 30-day mortality (AUC = 0.635, 95% CI 0.592–0.679) and for 1-year mortality (AUC = 0.666, 95% CI 0.631–0.701) exhibited the highest predictive value among the considered severity of illness scores. Furthermore, we applied the Spearman correlation analysis to examine the relationship between SOFA, OASIA, LODS, SAPS II score, and SII. According to Supplementary Tables S5–S8, we find that SOFA score and OASIS score show a positive correlation with SII (Spearman correlation = 0.085 and 0.070, respectively). LODS and SAPS II scores do not correlate with SII.

### 3.3 Subgroup analysis

**Figure 6:**
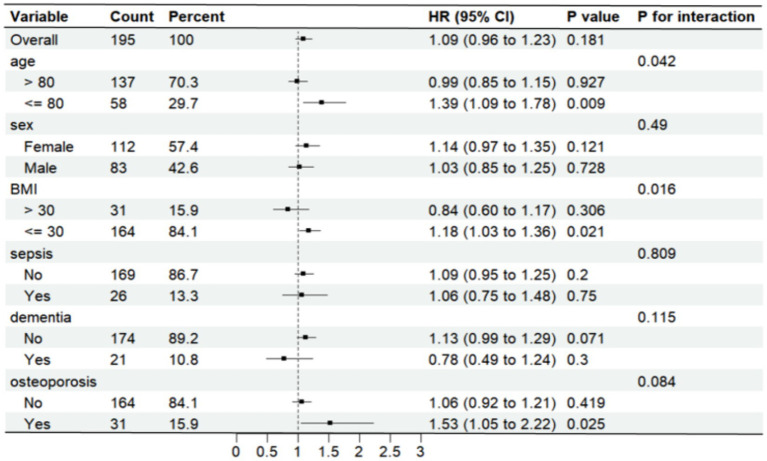
Forest plots displaying hazard ratios for 30-day mortality across different subgroups. HR, hazard ratio; CI, confdence interval; BMI, body mass index.

**Figure 7:**
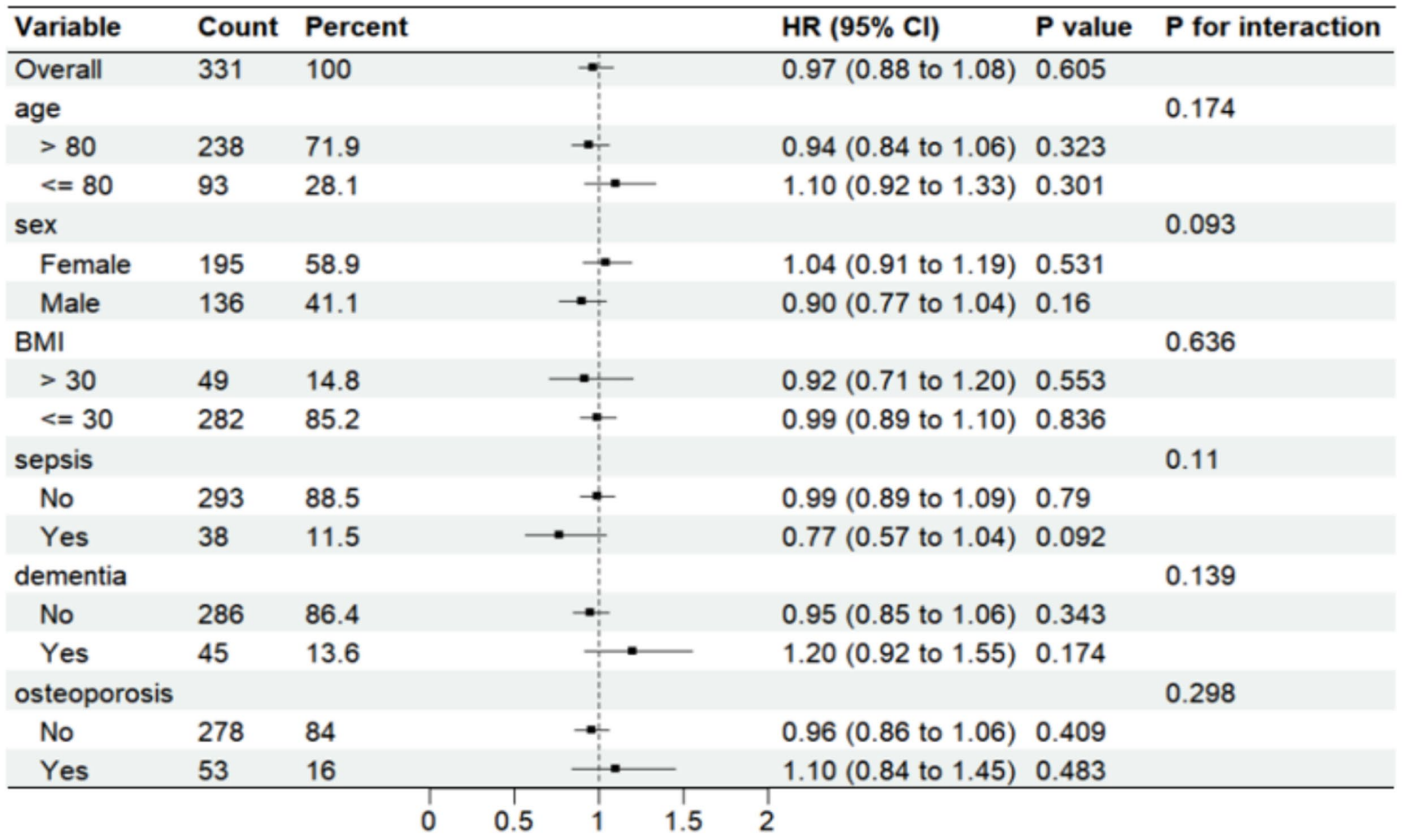
Forest plots displaying hazard ratios for 1-year mortality across different subgroups. HR, hazard ratio; CI, confdence interval; BMI, body mass index.

Further analysis was made of SII risk stratification values for primary endpoints, including age, sex, body weight index, sepsis, dementia, and osteoporosis, in multiple subgroups of the enrolled patients (Figures 6, 7). The results demonstrated that SII had no significant interaction with stratified variables such as sex, dementia, sepsis, and osteoporosis on 30-day mortality (all P for interactions were > 0.05). Similarly, SII also had no significant interaction with stratified variables such as age, sex, BMI, dementia, sepsis, and osteoporosis on 1-year mortality (all P for interactions were > 0.05). The SII was profoundly linked to an increased risk of 30-day mortality in critically ill elderly patients with hip fracture subgroups of those aged ≤80 years [HR (95% CI) 1.39 (1.09–1.78)], those with BMI ≤ 30 kg/m2 [HR (95% CI) 1.18 (1.03–1.36)], and those with osteoporisis [HR (95% CI) 1.53 (1.05–2.22)]. However, SII did not significantly affect subgroups of critically ill elderly patients with hip fracture in terms of 1-year mortality after hospital admission.

## 4 Discussion

The study investigated the relationship between the SII and clinical results in critically ill elderly patients with hip fractures using the MIMIC-IV database. Analysis found a strong connection between a raised SII and increased 30-day and 1-year mortality rates from all causes in critically ill elderly adults with hip fracture. The SII remained significantly correlated with all-cause 30-day and 1-year mortality after admission, even when accounting for confounding risk variables. Therefore, the SII could function as a crucial tool for clinical decision-making and potentially stand as an individual risk indicator in critically ill elderly patients with hip fractures.

Patients with elevated SII values exhibited increased heart rate, respiration rate, anion gap, BUN, SOFA scores, heparin utilization, and lower BMI, total bilirubin, chloride, and calcium, which suggests that these indicators are strongly linked to the negative outcomes of critically ill elderly patients, as shown in various prior research studies. For instance, prior research demonstrated that patients with a lower BMI often have concomitant complications such as malnutrition, hypoproteinemia, and osteoporosis, and they have a higher one-year postoperative mortality rate and poorer functional outcomes (24–26).Furthermore, patients with higher cardiac and respiratory rates had an increased likelihood of in-hospital mortality (27). Another research investigation demonstrated that those with a significantly higher plasma anion gap have a poorer clinical outcome and an increased likelihood of dying while in the hospital (28). Biochemical ionic disturbances, blood urea nitrogen, and total bilirubin levels on admission have also been associated with a poorer prognosis for hip fracture (29). The SOFA score, a metric for evaluating disease severity in ICU patients, has been linked to an unfavorable outcome in fracture patients (30). Heparin utilization reflects the coagulation status of the patient population, which tends to be at higher risk for VTE and DVT (31).

The SII, consisting of neutrophil counts, lymphocyte counts, and platelet counts, is proposed as a viable marker for preoperative status assessment, surgical trauma, and postoperative complications in the field of orthopedics (10–12). Numerous clinical studies have delved into the association between SII and the incidence as well as mortality rates of fractures and complications across the general populace and diverse patient categories. Zhang et al. (32) reported that there exists an association between increased SII and an increased vulnerability to systemic inflammation as well as osteoporosis in middle-aged and elderly populations. Fang et al. (33) found that monitoring changes in SII could play a role in predicting osteoporotic fractures. And for patients with hip fractures, Zeng et al. (34) reported that SII could potentially be a useful indication for predicting preoperative DVT development in individuals with intertrochanteric fractures of the femur. Moldovan et al. (11) found that SII was strongly associated with surgical trauma suffered by an elderly population with hip fractures. Bala (35) found that short-term functional projections in hip fracture patients who undergo hemiarthroplasty could potentially be foreseen through the utilization of SII. Further, a prospective study involving 290 participants showed that in elderly patients who underwent hip fracture surgery, a strong association was observed between SII and increased mortality due to any cause (5). In addition, several studies have demonstrated the role of SII in reflecting systemic inflammation and disease severity (36, 37), particularly the diagnostic significance of SAPSII in orthopedic diseases (38, 39). These studies suggested that SII showed potential for predicting clinical outcomes in critically ill elderly individuals with hip fractures.

The precise biological mechanisms that connect the SII with the occurrence of morbidity and mortality following hip fracture in older individuals remain unidentified. The possible pathways may be related to body immune dysfunction and systemic inflammatory activation after a hip fracture. The underlying mechanisms likely include a sequence of inflammatory cytokines and chemokines, which stem from the dysfunction of lymphocytes within the immune reaction, resulting in the accumulation of neutrophils and macrophages (40, 41). Also, injury, infection, or ischemia can trigger a defensive inflammatory response, leading to an increase in platelets (42). The nature of reduced physiological reserve in elderly patients makes them more vulnerable to post-injury release of aggressive cytokines (TNF-a, IL-6, and IL-1β, etc.), as well as the lack of sufficient anti-invasive mediators to counterbalance their negative effects (3). After a hip fracture, the hip fracture-induced plasma mitochondrial dried DNA (mtDNA) release leads to a systemic hyperinflammatory response and lung damage via activating the TLR9/NF-KB pathway, a process that has also been termed the “inflammatory storm” (43–46). Subsequent surgical trauma is rapidly exacerbated by sterile systemic invasive reactions and damage to vascular endothelial cells (47). In addition, pro-inflammatory cytokines can mediate oxidative stress injury, trigger osteoclasts, and enhance bone resorptive properties, which can gradually lead to bone remodeling and even osteoporosis (48). The inflammatory response increases the body’s pro-inflammatory mediators, aggravating excessive inflammation, and the excessive inflammatory factors pass through the bloodstream to the lungs, heart, and other distant organs, aggravating the damage to the heart, lungs, and other organs and making it easier to trigger coagulation dysfunction, respiratory infections, and other symptoms, which can lead to the failure of other organ functions. Inflammatory co-morbidities are accompanied by a progressive collapse of the immune system, which leads to negative clinical consequences (49).

Presently, there is little literature exploring the correlation between the SII and severely ill individuals. Alsabani et al. (50) discovered that in critically ill patients, SII was linked to a higher likelihood of undergoing prolonged hospitalization after orthopedic surgical procedures; IMA et al. noted that in critically ill patients undergoing extracorporeal coronary artery bypass grafting, the preoperative SII was found to be an indicator for postoperative complications, including cardiac arrest and acute myocardial infarction, as well as associated with an increased risk of mortality (51); research utilizing the MIMIC database revealed a substantial link between the SII and sepsis-related hospital mortality among critically ill individuals presenting with toxemia (52). However, in our ICU hip fracture patient group, the study found that SII emerged as a considerable predictor of heightened mortality among critically ill patients. Moreover, in the case of hip fracture, a common condition with significant morbidity and mortality, our research indicates that the SII might be beneficial for pinpointing high-risk patients before surgery and potentially help in minimizing severe future complications.

Furthermore, this study further conducted a detailed analysis of the risk stratification across various subpopulations. The stratified analysis revealed a uniform predictive strength of SII concerning 1-year mortality that was consistent in critically ill individuals with hip fracture. However, no association was discovered between the SII and 30-day or 1-year mortality in individuals with sepsis or dementia at baseline. The observed trend could stem from a reversed causal relationship, suggesting that individuals with specific co-existing conditions are more inclined to have received suitable treatment or embraced good lifestyle practices (53, 54). Moreover, the investigation revealed that the organism’s condition of 30-day after hospital admission, evaluated through the SII, exhibited a heightened predictive significance in individuals of those aged ≤80 years [HR (95% CI) 1.39 (1.09–1.78)], those with BMI ≤ 30 kg/m2 [HR (95% CI) 1.18 (1.03–1.36)], and those with osteoporisis [HR (95% CI) 1.53 (1.05–2.22)]. This suggests that treating osteoporosis could greatly affect the predictive accuracy of SII for all-cause mortality. Past research has shown a strong connection between the overall immunological and inflammatory condition of the body and osteoporosis, possibly due to the direct or indirect impact of immune cells on the functions of bone cells (55).What’s more, we confirm the association between SII and mortality is more pronounced in a population of critically ill hip fracture patients with a lower BMI. Another interesting finding of the present study was that patients with higher SII were younger, and the link between SII and all-cause death appeared to be more pronounced in younger patients. Our findings suggest that clinicians should offer equal attention to younger patients due to their potentially greater death rate rather than focusing solely on older patients with more comorbidities. The study revealed a positive association between SII and 30-day mortality and 1-year mortality among severely ill individuals who had suffered from hip fractures, but this correlation is a non-linear relationship, suggesting that SII could serve as an effective instrument for identifying high mortality risk in this patient population. Therefore, early detection of SII, screening of high-risk groups, and improved management of SII are crucial to decreasing significant negative clinical results in the future. In a word, the evaluation outcomes suggest that the SII should not be employed as a solitary diagnostic instrument. Its application should complement other medical and laboratory assessments to provide a thorough evaluation of a patient’s health condition and enhance the risk appraisal for occurrences like mortality subsequent to a hip fracture within clinical settings.

This study’s primary strength lies in the compelling evidence we present, establishing an elevated SII as a significant standalone predictor of higher mortality rates among critically ill elderly patients with hip fracture. Nevertheless, there are certain constraints to consider in our investigation. Firstly, this study was unable to prove causality because of its retrospective methodology. Despite utilizing multivariate adjustments and subgroup analysis, residual variables could still impact clinical outcomes. Important variables that could affect the results, like the exact type of hip fracture and whether it was treated with surgery, were not available in this database. Secondly, the research was executed exclusively within a single-center setting, concentrating on elderly participants with an elevated risk of hip fracture; therefore, the study may contain selection bias. Third, the study solely assessed the baseline SII index at the initial hospitalization. There was no significant change in the SII index before and after hospitalization. Hence, the prognostic value of alterations in the SII should be assessed in upcoming research as well. Fourthly, our data were extracted based on the MIMIC-IV database and the follow-up period commenced on the admission date and ended on the day of death, but the study fail to address potential changes in care over the years or clarify the number of patients treated each year of follow-up. Fifthly, our study lacked some blood indices, including cytokines such as interleukins and high-sensitivity C-reactive protein, which precluded us from exploring the association between the SII and conventional inflammatory markers. Consequently, the necessity arises for additional multicenter prospective studies aimed at validating the relevance of SII in predicting the long-term outcome among aged, critically ill patients with hip fractures.

## 5 Conclusion

Our findings expanded the applicability of the SII to older patients with hip fractures who are critically ill. The study showed that the SII could serve as a valuable tool for assessing the risk of all-cause mortality within 30 days and 1 year in this patient population. Observing the SII may be beneficial in decision-making and disease management in clinical settings. Additional research is required to further confirm the potential of SII to enhance clinical prognosis in the future.

## Data availability statement

The original contributions presented in the study are included in the article/Supplementary materials, further inquiries can be directed to the corresponding authors.

## Ethics statement

The studies involving humans were approved by the MIT Computational Physiology Laboratory. The studies were conducted in accordance with the local legislation and institutional requirements. The participants provided their written informed consent to participate in this study.

## Author contributions

Z-JL: Conceptualization, Data curation, Formal analysis, Funding acquisition, Project administration, Supervision, Writing – original draft, Writing – review & editing, Resources. G-HL: Writing – original draft, Data curation, Methodology, Validation. J-XW: Writing – original draft, Data curation, Investigation. Z-HM: Data curation, Investigation, Writing – original draft. K-YY: Software, Visualization, Writing – review & editing. C-LS: Project administration, Software, Supervision, Visualization, Writing – original draft, Writing – review & editing. Z-XS: Project administration, Resources, Software, Supervision, Validation, Visualization, Writing – original draft, Writing – review & editing.
