# Supplementary material for: Prognostic value of the systemic immune-inflammation index in critically ill elderly patients with hip fracture: evidence from MIMIC (2008–2019)
Source: Front Med (Lausanne). 2024 May 30;11:1408371. doi: 10.3389/fmed.2024.1408371 (PMC11169710; doi:10.3389/fmed.2024.1408371)
Supplement: Supplementary file 1 [file Table_1.DOCX]

Supplementary Material

# Supplemental Table 1. Missing values of included individuals.

| Variable | Missing number (%) |
| --- | --- |
| Age（year） | 0 |
| Sex:male | 0 |
| BMI | 38 |
| Temperature (℃) | 2 |
| Heart rate (beats/min) | 7 |
| Respiratory rate (breaths/min) | 7 |
| SBP (mmHg) | 5 |
| DBP (mmHg) | 5 |
| MBP (mmHg) | 5 |
| SpO2 (%) | 2 |
| Diabetes mellitus | 0 |
| Rheumatic Disease | 0 |
| COPD | 0 |
| PE | 0 |
| DVT | 0 |
| Dementia | 0 |
| Coronary heart disease | 0 |
| Osteoporosis | 0 |
| Sepsis | 0 |
| CKD | 0 |
| Pneumonia | 0 |
| Cerebral infarction | 0 |
| Hypertension | 0 |
| Laboratory tests | 0 |
| Aniongap(mEq/L) | 0 |
| CK-MB(ng/ml) | 25 |
| BUN (mg/dL) | 0 |
| ALP（U/L） | 56 |
| Bicarbonate(mmol/L) | 0 |
| Totalbilirubin(mg/dL) | 19 |
| Sodium(mmol/L) | 14 |
| Chloride(mmol/L) | 14 |
| Calcium(mmol/L) | 14 |
| Potassium(mmol/L) | 0 |
| PT(s) | 0 |
| PTT(s) | 0 |
| PT INR | 39 |
| Creatinine(mg/dL) | 0 |
| WBC counts(109/L) | 0 |
| Platelet counts (109/L) | 0 |
| Lymphocyte counts(109/L) | 0 |
| Neutrophil counts(109/L) | 0 |
| Monocyte count(109/L) | 0 |
| Hemoglobin(g/dL) | 10 |
| Hematocrit(%) | 0 |
| Heparin | 0 |
| Mechanical ventilation | 0 |
| SII | 0 |
| SOFA score | 15 |
| SAPS II | 16 |
| LODS | 36 |
| OASIS | 27 |

# Supplemental **FIGUR 1** Receiver operating characteristic (ROC) curve of SII

#
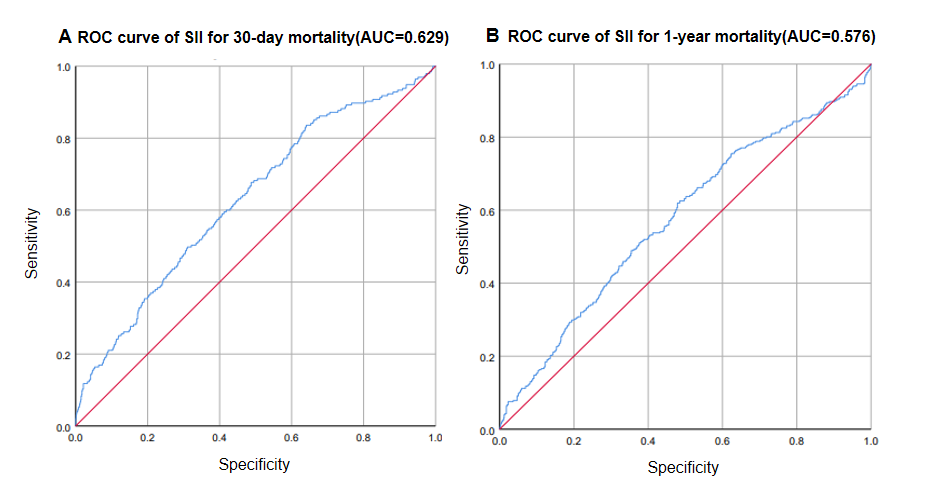


# Supplemental Table 2. Binary logistic regression analysis of the factors influencing all-cause death of the study population.

| Variables | OR | Lower 95% CI | Upper 95% CI | P-value |
| --- | --- | --- | --- | --- |
| Age | 1.05 | 1.023 | 1.078 | <0.001 |
| Sex: male | 0.624 | 0.445 | 0.874 | 0.006 |
| Coronary heart disease | 0.929 | 0.628 | 1.374 | 0.713 |
| Osteoporosis | 1.288 | 0.794 | 2.089 | 0.305 |
| Sepsis | 0.268 | 0.143 | 0.503 | <0.001 |
| Bicarbonate | 1.014 | 0.964 | 1.067 | 0.581 |
| Aniongap | 0.983 | 0.928 | 1.042 | 0.573 |
| Hemoglobin | 0.826 | 0.688 | 0.992 | 0.041 |
| Creatinine | 1.015 | 0.81 | 1.272 | 0.897 |
| aalcium | 0.936 | 0.886 | 0.988 | 0.017 |
| SII | 1.176 | 1.068 | 1.295 | 0.001 |
| WBC | 1.001 | 0.96 | 1.043 | 0.975 |

# Supplemental Table 3. Receiver operating characteristic (ROC) curve of severity of illness scores for 30-day mortality

| Variables | AUC | Lower 95% CI | Upper 95% CI | P-value |
| --- | --- | --- | --- | --- |
| OASIS | 0.622 | 0.578 | 0.667 | <0.001 |
| LODS | 0.625 | 0.581 | 0.668 | <0.001 |
| SOFA | 0.559 | 0.510 | 0.608 | 0.011 |
| SAPS II | 0.635 | 0.592 | 0.679 | <0.001 |

# Supplemental Table 4. Receiver operating characteristic (ROC) curve of severity of illness scores for 1-year mortality

| Variables | AUC | Lower 95% CI | Upper 95% CI | P-value |
| --- | --- | --- | --- | --- |
| OASIS | 0.629 | 0.593 | 0.666 | <0.001 |
| LODS | 0.651 | 0.615 | 0.687 | <0.001 |
| SOFA | 0.503 | 0.463 | 0.542 | 0.883 |
| SAPS II | 0.666 | 0.631 | 0.701 | <0.001 |

# Supplemental Table 5. Spearman Correlation Analysis between SII and SOFA score

|  |  | SII | SOFA socre |
| --- | --- | --- | --- |
| SII | Spearman correlation | 1 | 0.085** |
|  | P-value |  | 0.008 |
| SOFA score | Spearman correlation | 0.085 | 1 |
|  | P-value | 0.008 |  |
| *p<0.05**p<0.001 | | | |

# Supplemental Table 6. Spearman Correlation Analysis between SII and OASIS score

|  |  | SII | OASIS socre |
| --- | --- | --- | --- |
| SII | Spearman correlation | 1 | 0.070* |
|  | P-value |  | 0.027 |
| OASIS score | Spearman correlation | 0.070* | 1 |
|  | P-value | 0.027 |  |
| *p<0.05**p<0.001 | | | |

**Supplemental Table 7. Spearman Correlation Analysis between SII and LODS score**

|  |  | SII | LODS socre |
| --- | --- | --- | --- |
| SII | Spearman correlation | 1 | 0.050 |
|  | P-value |  | 0.115 |
| LODS score | Spearman correlation | 0.050 | 1 |
|  | P-value | 0.115 |  |
| *p<0.05**p<0.001 | | | |

**Supplemental Table 8. Spearman Correlation Analysis between SII and SAPS II score**

|  |  | SII | SAPS II socre |
| --- | --- | --- | --- |
| SII | Spearman correlation | 1 | 0.032 |
|  | P-value |  | 0.318 |
| SAPS II score | Spearman correlation | 0.032 | 1 |
|  | P-value | 0.318 |  |
| *p<0.05**p<0.001 | | | |
